# Acceptability of the “MOVEdiabetes” physical activity intervention in diabetes primary care settings in Oman: findings from participants and practitioners

**DOI:** 10.1186/s12889-020-09029-1

**Published:** 2020-06-08

**Authors:** Thamra S. Alghafri, Saud M. Al Harthi, Fatma Al-Ajmi, Yahya Al-Farsi, Angela M. Craigie, Elaine Bannerman, Annie S. Anderson

**Affiliations:** 1 Directorate General of Health Services, Ministry of Health, Muscat, Oman & University of Dundee, PO Box 2723, Postal Code 112 Muscat, Oman; 2 Directorate General of Health Services, Ministry of Health, Muscat, Oman; 3 Department of Family Medicine and Public Health, College of Medicine and Health Sciences, Sultan Qaboos University, Muscat, Oman; 4 Centre for Public Health Nutrition Research, Ninewells Hospital and Medical School, University of Dundee, Dundee, UK; 5 Global Academy of Agriculture & Food Security, The Royal (Dick) School of Veterinary Studies, University of Edinburgh, Edinburgh, UK

**Keywords:** Physical activity, Type 2 diabetes, Primary health care, MOVEdiabetes, Intervention, Oman, Recruitment and retention

## Abstract

**Background:**

Adequate physical activity (PA) is considered essential in diabetes management. However, evidence on the best method of promoting PA within diabetes care is inconclusive. The current work identifies perceptions on the acceptability of Intervention Group Participants (IGP) and Project Officers (POs) about the “MOVEdiabetes” intervention programme aimed at increasing PA in adults with type 2 diabetes in Oman (a retrospectively registered trial).

**Methods:**

The “MOVEdiabetes” programme (PA consultations, pedometers and WhatsApp messages) was delivered by the POs (primary health care practitioners) in four primary care centres within a one-year cluster randomised control trial. Recruitment and retention were measured from trial attendance records. Programme satisfaction, appropriateness, and content suitability were assessed using exit surveys for both the IGP (interview based) and POs (self-administered). Open text questions on perceptions to the study programme were also included.

**Results:**

Participants were randomised to an intervention group (IG, *n* = 122) or comparison group (CG, *n* = 110). The overall retention rate at three and 12 months was 92.7% [110(90.2%) IG vs 105(95.5%) CG] and 75% [82(67.2%) IG vs 92(83.6%) CG] respectively. Most (*n* = 14, 87.5%) POs and more than half (*n* = 49, 59.8%) IGP perceived the programme as very appropriate and many reported that they were “quite/ very satisfied” with the programme (*n* = 16, 100% PO’s and *n* = 71, 86.6% IGP). Two thirds (*n* = 55, 66.0%) of IGP were very/quite likely to recommend the programme to others. PA consultations, use of pedometers and Whatsapp messages were well perceived by all. Participants recommended the inclusion of dietary advice and PA promotion for the general public. Exploring PA facilities within the community was suggested by POs.

**Conclusions:**

The “MOVEdiabetes” programme achieved a high retention rate and was perceived as satisfactory and appropriate. Results from this study suggest that it is worthwhile exploring the use of the “MOVEdiabetes” programme in clinical practice and further community links.

**Trial registration:**

International Standard Randomised Controlled Trials No: ISRCTN14425284. Registered retrospectively on 12th April 2016.

## Background

Despite the strong evidence base for the inclusion of PA in the clinical management of diabetes [1], the majority of PA interventions have been performed in a controlled research setting, using resource intensive methods of short duration and limited follow-up [2]. Minimal information is available on how acceptable these interventions are when adapted and implemented within everyday practice [3].

Despite the reported barriers to promoting PA in clinical settings such as a lack of time, training and assessment tools [4], some methods to facilitate and support behavioural changes regarding PA in primary care have proven to be effective in several studies in Western cultures [5–7]. Current research in this area includes understanding components of PA interventions, barriers and motivators and effective methods of intervention delivery. However, few studies have examined the perceived appropriateness, suitability of interventions and implications of trial findings for roll out within local settings.

Moreover, to address cultural, social and clinical differences, it is important to evaluate the acceptability and appropriateness of transferring evidence from the West to the Arab world. Evaluating these aspects is important for further programme development and improvement, ensuring accountability to stakeholders and helping others set up similar services.

The use of process evaluations to guide the translation of research findings for effective clinical practice is increasing. However, a lack of consistent reporting of the evaluation findings remains an issue [8, 9].

Due limited evidence, in the Arabic world and Oman, series of studies were structured to provide formative evidence to inform a PA behavioural intervention within diabetes primary care in Oman. Results from formative work that has informed the design of a PA intervention named “MOVEdiabetes” have been published in several recognized international journals [4, 10–12]. To follow, the “MOVEdiabetes” intervention examined the impact of a multi-component intervention/trial (PA consultations, pedometers and WhatsApp phone messages) to increase PA in inactive adults with T2D in Oman. This paper describes the perceptions of the participants in the intervention group (IG) and all the project officers (POs) (health care practitioners in primary health care), of the “MOVEdiabetes” study in order to establish if the intervention (published elsewhere) was acceptable [10, 13].

## Methods

The study was a 1 year (April 2016 to June 2017) cluster randomized controlled trial of the “MOVEdiabetes” intervention versus usual care. The trial aimed at evaluating the effectiveness of the intervention on changes in PA levels (primary outcome), anthropometric (weight and BMI), and cardio-vascular outcome at 12 months from baseline [10]. Notably, the published “MOVEdiabetes” trial adhered to the CONSORT guidelines [13]. Health centres were randomised to deliver either the intervention (IG, *n* = 4) or usual care in the comparison group (CG, *n* = 4). The intervention group received the “MOVEdiabetes” personalised PA consultations, a pedometer (YAMAX Digi-walker SW-200) to measure weekly step counts and monthly WhatsApp support messages. Intervention delivery was undertaken by all members of the POs except for conducting the consultations which were confined to the dietitians only (maximum 20 min) on three occasions (0, 4 and 8 weeks). Participants were asked to set individual goals and complete daily (personal diaries) step counts which were to be submitted to the POs in their respective health centres at 3 and 12 months follow up shared through the WhatsApp phone application. Monthly standardised PA motivational messages, coinciding with religious and international occasions [such as Ramadhan, breast feeding day, cancer awareness day, and healthy lifestyle awareness day were all delivered through the WhatsApp telephone application [10].

The “MOVEdiabetes” intervention was effective in increasing PA levels, reducing sitting time and increasing the likelihood of meeting WHO PA recommendations in adults with T2D attending their routine diabetes primary care clinics over 12 month. Additionally, the intervention showed potentially protective cardiovascular effectiveness in reducing blood pressure and triglycerides levels [13].

The POs were required to undertake short meetings with the PI last week of every month to discuss attendance sheets, issues with the PA consultations, and participant appointment slots. Equally important, every effort was made to give the participants convenient appointments and reschedule appointments when needed. Additionally, the smart phones (specific for the “MOVEdiabetes” study) were used to facilitate the communication between the POs and PI. The WhatsApp telephone application (in addition to its use for intervention purposes) and phone calls were used throughout the study period to manage the daily logistics and administrative queries.

Worth mentioning, the ethical approval for this study was obtained from the Regional Research Committee in Muscat, Oman Ministry of Health within the overall “MOVEdiabetes” study.

### Details on measures/assessment instruments

Two questionnaires guided by some of the key components on program implementation, maintenance and fidelity, outlined by Linnan and Steckler [14] were developed to assess POs’ and IG participants’ perceived acceptability of the programme. The questions were in the format of 5-point Likert scales that included 11 items for participants and eight items for POs. Potential responses were anchored with a scale that ranged from positive to negative perceptions to the items in the questionnaires (Tables 1 and 2). Additionally, a number of open-ended questions, were included to explore perceptions from the POs and participants on areas were more information was required, challenges and general comments. Both surveys were developed to be completed at the end of trial (exit survey) (see Supplementary files 1 and 2).

**Table 1:** Intervention group participants’ responses to the exit survey (completed 12 months follow up *n* = 82)

Questions	Responses n(%)				
Overall, how satisfied were you with the “MOVEdiabetes” project?	Very dissatisfied	Quite dissatisfied	Neither satisfied nor dissatisfied	Quite satisfied	Very satisfied
	0	2 (2.4)	9 (11)	36 (43.9)	35 (42.7)
Do you feel you received enough information about the project at the start?	Far too little	Not enough	Sufficient information	More information than was necessary	Far too much information
	2 (2.4)	5 (6.1)	24 (29.3)	36 (43.9)	15 (18.3)
Did you have enough opportunity to ask questions during the project?	Not at all	Rarely	Every once in a while	Sometimes	Very often
	0	0	2 (2.4)	17 (20.7)	63 (76.8)
Were your questions answered to your satisfaction?	Not at all	Rarely	Every once in a while	Sometimes	Yes, completely
	0	1 (1.2)	11 (13.4)	44 (53.4)	26 (31.7)
How likely are you to recommend “MOVEdiabetes” to other people?	Very unlikely	Quite unlikely	Neither likely nor unlikely	Quite likely	Very likely
	0	9 (11)	18 (23)	10 (12)	45 (54)
How did you find coming up to the health centre for your appointments?	Very difficult	Quite difficult	Neither easy nor difficult	Quite easy	Very easy
	0	1 (1.2)	8 (9.8)	28 (34.1)	45 (54.9)
Having taken part, do you think this program is appropriate in diabetes care?	No, not at all appropriate	Quite inappropriate	Neither appropriate or inappropriate	Quite appropriate	Very appropriate
	0	2 (2.4)	10 (12.2)	21 (25.6)	49 (59.8)
Is your change in physical activity behaviour acceptable?	No change, not at all	Very Little change	Not sure	Somewhat	To a great extent
	2 (2.4)	9 (11)	14 (17)	30 (36.6)	27 (32.9)
Please rate the consultations you received	Very poor	Poor	Acceptable	Good	Very good
Content	0	0	11 (13.4)	24 (29.3)	47 (57.3)
Relevance	0	0	4 (4.9)	15 (18.3)	63 (76.8)
Duration per consultation	0	0	3 (3.7)	7 (8.5)	72 (87.8)
Frequency	0	0	2 (2.4)	15 (18.3)	65 (79.3)
Please rate using pedometers	Very poor	Poor	Neither poor nor good	Fairly good	Very good
Length of device use	6 (7.3)	16 (19.5)	20 (24.4)	11 (13.4)	29 (35.4)
Importance to diabetes management	0	0	15 (18.3)	21 (25.6)	46 (56.1)
Wearing it (putting on and off)	0	0	15 (18.3)	17 (20.7)	50 (61)
Usefulness	0	0	6 (7.3)	14 (17.1)	62 (75.6)
Please rate the WhatsApp communication you received	Very poor	Poor	Neither poor nor good	Fairly good	Very good
Content	0	0	9 (11)	3 (3.7)	70 (85.4)
Relevance	0	0	6 (7.3)	4 (4.9)	72 (87.8)
Time required	0	0	9 (11)	13 (15.9)	60 (73.2)
Frequency of messages	0	0	3 (3.7)	13 (15.9)	66 (80.5)
Supportiveness	0	0	1 (1.2)	11 (13.4)	70 (85.4)

**Table 2:** Project officers’ perceptions of acceptability of the “MOVEdiabetes” project at 12 months (exit survey) (*n* = 16)

Questions	Responses				
Overall, how satisfied were you with the “MOVEdiabetes” project?	Very dissatisfied	Quite dissatisfied	Neither satisfied nor dissatisfied	Quite satisfied	Very satisfied
	0	0	0	3 (18.8)	13 (81.2)
Do you feel you received enough training about the project at the start?	Far too little	Not enough information	Sufficient information	More information than was necessary	Far too much information
	0	0	10 (62.5)	3 (18.8)	3 (18.8)
Did you have enough opportunity to ask questions during the project?	Not at all	Rarely	Every once in a while	Sometimes	Very often
	0	0	0	2 (12.5)	14 (87.5)
Were your questions answered to your satisfaction?	Not at all	Rarely	Every once in a while	Sometimes	Yes, completely
	0	0	0	6 (37.5)	10 (62.5)
Please rate the consultations you conducted^a^	Very poor	Poor	Acceptable	Good	Very good
Content	0	0	0	0	4 (100)
Relevance	0	0	0	0	4 (100)
Frequency	0	0	0	0	4 (100)
Please rate the use of pedometers as physical activity self-monitoring tool	Very poor	Poor	Acceptable	Good	Very good
Usefulness	0	0	0	0	16 (100)
Relevance to diabetes management	0	0	0	0	16 (100)
Please rate the WhatsApp communication you were involved in	Very poor	Poor	Acceptable	Good	Very good
Content	0	0	0	0	16 (100)
Relevance	0	0	0	0	16 (100)
Time required	0	0	0	0	16 (100)
Frequency of messages	0	0	0	3 (18.8)	13 (81.3)
Having taken part, do you think this programme is appropriate/suitable in diabetes primary care?	Not at all appropriate/suitable	Not very appropriate/suitable	Not sure	Quite appropriate/suitable	Very appropriate/suitable
The “MOVEdiabetes” study overall	0	0	0	2 (12.5)	14 (87.5)
Consultations	0	0	0	3 (18.8)	13 (81.3)
Pedometers	0	0	0	5 (31.3)	11 (68.8)
Personal PA diaries	0	0	0	4 (25)	12 (75)
WhatsApp	0	0	0	6 (37.5)	10 (62.5)

^a^The face to face personalised PA consultations were all carried out by the dietitians only

To maximise content validity for selection of items/questions, the revision process of the questionnaires involved assessment by the authors of this study (EB, AMC and ASA) and two independent PA researchers from Oman (SMA, FA, and YF) and a subsequent revision in light of their feedback by TSA. Prior to field administration, an internal pilot testing with a convenience sample of adults with T2D (*n* = 10) was carried out. Minor changes were made to ensure clear understanding and smooth flow of the questions including re-arranging and locating the questions and responses.

### Participants’ exit survey

The 11-item survey was an interviewer-led questionnaire administered by an independent nurse/researcher who interviewed the participants who completed 12 months study follow up (*n* = 82) and recorded their responses. All participants who attended the 12-month visit were approached and invited to complete the survey.

The items explored overall satisfaction of the project (from ‘very dissatisfied’ to ‘neither satisfied nor dissatisfied’ to ‘very satisfied’), if information received regarding the project was enough (from ‘too little’ to ‘sufficient information’ to ‘too much information’), if they had enough opportunities to ask questions (from ‘not at all’ to ‘every once in a while’ to ‘very often’) and if answers to their questions were satisfactory (from ‘not at all’ to ‘every once in a while’ to ‘completely satisfactory’). Additionally, the survey included questions on the likelihood of recommending the intervention to others (from ‘very unlikely’ to ‘neither likely nor unlikely’ to ‘very likely’), accessibility to the health centres (from ‘very difficult’ to ‘neither easy nor difficult’ to ‘very easy’), and if the intervention was appropriate in diabetes care (from ‘not at all appropriate’ to ‘neither appropriate or inappropriate’ to ‘very appropriate’). Participants were also asked if they perceived their PA behaviour change to be acceptable (from ‘not at all’ to ‘not sure’ to ‘very acceptable’).

To follow, participants were asked to rate each intervention component (face to face consultations, pedometers and use of WhatsApp) from a range of five options from ‘very poor’ to ‘acceptable’ to ‘very good’ (Table 1). The consultations were rated for their content, relevance, duration and frequency. Pedometers on the other hand were rated for length of the device use, importance to diabetes care, longevity, and usefulness. Finally, WhatsApp communications were rated for their content, relevance, time required, frequency of messages, and supportiveness (Table 1).

Four open ended questions queried participants’ perceptions of: a) aspects of the project where more information was needed, b) challenges of taking part in the project, c) barriers to increasing physical activity behaviour, and d) general comments.

### Project officers’ exit survey

The POs were recruited from existing health care providers (doctors/nurses/dietitians/health educators) involved in diabetes primary care. Project officers received study specific training on the recruitment procedures, screening the participants, recording outcome measurements, and delivering the “MOVEdiabetes” intervention in intervention health centres. A self-reported eight-item (five-point Likert scale) based questionnaire was completed by all POs at the end of the “MOVEdiabetes” study. Questions included overall satisfaction with the intervention (from ‘very dissatisfied’ to ‘neither satisfied nor dissatisfied’ to ‘very satisfied’), if training received prior to the intervention delivery was enough (from ‘far too little’ to ‘sufficient information’ to ‘far too much’), if they had opportunities to ask questions (from ‘not at all’ to ‘every once in a while’ to ‘very often’) and if the answers to their questions were satisfactory (from ‘not at all’ to ‘every once in a while’ to ‘completely’). An additional question was included on the appropriateness of the intervention in diabetes care (from ‘not at all appropriate’ to ‘not sure’ to ‘very appropriate’). Also, individual components of the “MOVEdiabetes” intervention namely use of pedometers, WhatsApp and PA consultations, were rated (from ‘very poor’ to ‘acceptable’ to ‘very good’) by POs in terms of content, relevance, and frequency of the PA consultations. For use of pedometers, ratings were on their usefulness and relevance. WhatsApp communications on the other hand, were rated for content, relevance, time required and frequency of messages. Finally, a general question on the suitability of each of the intervention components in diabetes care was included (‘not at all suitable’ to ‘not sure’ to ‘very suitable’). Additionally, open-ended questions were used for POs to document their perceptions on topics which required more information, for example, any challenges to delivering the intervention and if they had any further comments.

### Analysis

Mixed methods were used to analyse data on acceptability: Frequency tables were used to present response numbers (n) and proportions (%) for all items in the questionnaires using IBM SPSS Statistics for Windows (V 22).

Open ended questionnaire responses were transcribed verbatim and analysed using thematic content analysis [15]. Initial responses were read several times by the principal investigator (TSA) followed by open coding, grouping and categorizing data according to emerging themes. A coding scheme was then developed based on the major recurring themes. Themes were cross-checked by another independent researcher (SA) and areas of contradiction were discussed and adjusted. A final revision was carried out by one of the authors (YF) as a further measure of inter-rater reliability.

## Results

### Recruitment and retention

Out of 232 enrolled participants (117 IG, 110 CG) completed measurements at baseline. Overall retention was 92.7% [110(90.2%) IG vs 105(95.5%) CG] at3 and 75% [82(67.2%) IG vs 92(83.6%) CG] at 12 months.

### Participant exit survey

Overall, results presented in Table 1 indicate positive response. All participants in the IG who completed the 12 months visit (*n* = 82) responded to the exit questionnaire (38 male, 44 female). Most participants were ‘very satisfied’ (*n* = 35, 42.7%) or ‘quite satisfied’ (*n* = 36, 43.9%) with the project. Only 11% (*n* = 9) were ‘not sure’ and very few were ‘quite dissatisfied’ (*n* = 2, 2.4%).

Most participants felt the information received was ‘more than necessary’ or ‘sufficient information’ (43.9 and 29.3%, respectively) although a fifth of the participants thought it was ‘far too much’ (18.7%). In addition, participants reported positively to their opportunity to ask questions and satisfaction to the answers.

Overall, responses were largely positive with 66% indicating they were very/quite likely to recommend the project to others. Other participants were unsure (23%) and few were ‘very unlikely’ (11%).

Accessibility to respected health centres was very/quite easy to majority of the participants and most participants perceived the project as ‘very appropriate’ and ‘quite appropriate’ for use within local diabetes primary care.

### Perceptions on intervention response and components

Two thirds of the participants perceived their PA behaviour to have changed to a ‘great extent’ and ‘somewhat’. However, a third (30.4%) were ‘not sure’ or experienced ‘very little’ change or ‘no change’.

Responses about longevity were less positive (49% reported the longevity of the pedometers to be ‘good’ or ‘very good’, however most perceived their importance to diabetes management, wearing them and usefulness as ‘good’ to ‘very good’.

Except for use of pedometers (length of device use), no one responded ‘poor’ or ‘very poor’ to any of the other intervention components. Content, relevance, duration of the PA consultations and frequency were all perceived as ‘good’ to ‘very good’ by most of the participants. Additionally, responses about the WhatsApp monthly messages were all perceived as ‘fairly good’ to ‘very good’ by the majority of the participants.

### Project officers’ exit survey

Sixteen POs participated (2 male and 14 female) in intervention delivery and data collection across the eight randomly selected health centres: eight doctors, four nurses and 4 dietitians (Table 2). The PA consultations were delivered by the dietitians only. Overall responses were all positive. All POs were either ‘very satisfied’ or ‘quite satisfied’ with the project although three POs considered it excessive.

Opportunity to ask questions and answers were all well perceived by the majority of the POs. All the dietitians (*n* = 4) who delivered the face to face personalised PA consultations perceived the consultations as ‘very good’ for content, relevance and frequency. Additionally, all POs perceived the pedometers as useful and relevant to diabetes management. Content, relevance, and time required for activation of WhatsApp messages were perceived as ‘very good’. Perceptions on the frequency of WhatsApp messages were ‘very good’ by majority of POs.

Overall the intervention was perceived as ‘very appropriate’ or ‘quite appropriate’ by all POs. Moreover, suitability of all the “MOVEdiabetes” components (consultations, pedometers, personal PA diaries and WhatsApp messages) were perceived as ‘quite’ to ‘very suitable’ with no negative or neutral responses.

### Responses to the open questions

*Responses from the****participants?***

Responses to the open questions varied across the questions (see Supplementary file 3).

Overall, 43.9% of the participants expressed an interest in knowing more about types of exercises *“What type of exercise is suitable for patients with diabetes? P_HC1”*, the use of accelerometers “*What is the purpose of the accelerometers? P_HC1”,* and PA options in the presence of comorbidities *“I have glaucoma, can I exercise? P_HC2*”.

Only 25.6% participants responded to the question on challenges of taking part in this project. Two themes were identified as challenges of taking part in the project: long and exhausting measurement tools “*The questionnaires are too long and time-consuming P_HC4”* and lack of time for intervention delivery “*I don’t have time to attend the PA consultations P_HC2”.*

Barriers to increasing PA behaviour were identified by 43.9% participants. Main barriers were: hot weather *“It is too hot outside, I can’t walk P_HC4”*, lack of time *“I have no time for physical activity P_HC1”*, resources “*I don’t know where to go for physical activity P_HC4”,* and pain “*I can’t exercise, I have pain in my knees P_HC2”*.

Finally, general comments from 24.3% participants were themed as: inclusion of dietary advice *“I suggest to add diet advice P_HC2”*, project sustainability “*Keep the project, don’t stop P_HC3”,* and a similar project for all including children and the public *“Develop similar projects for children/public P_HC3 &P_HC1”*.

*Responses from the****POs?***

Notably, number/percentage of responses from the POs (*n* = 16) varied across the questions. Three quarters (75%, *n* = 12) of the POs provided responses to the open question on topics which required more information in the survey and are listed in full as verbatim quotes in Supplementary file 4. Among those who responded to this question, half of them reported that they required more information on the PA behaviour change techniques (BCT) “*We need more PA training especially on the behaviour change techniques PO1”,* and PA measurement tools “*More information is needed on the measurement tools or devices PO1”.*

Themes identified for challenges (*n* = 16) to delivering the intervention were categorised as physical challenges and logistical challenges including: “*No dedicated room/space PO3”,* “*Busy clinics PO2”,* “*Long questionnaires PO16”, “Managing appointments is difficult PO5”*, and “*Handling accelerometers is difficult PO10”.*

Themes to the general comments from the POs (*n* = 14) were related to sustaining the project “*WhatsApp Communications may be useful for future PA interventions PO8”* and *“Include PA in the Health information system PO3”.* Additionally, importance of identifying available PA facilities in communities was highlighted “*Implement this project in all health centres PO12” and “We need information on the available PA facilities in the nearby community PO5”.*

## Discussion

### Implications of study results on the current practice and future studies

This paper aimed to provide evidence on the perceived acceptability of the “MOVEdiabetes” study to POs and participants in the IG. Overall, the majority of the IG participants (who completed the 12 months study period) and all POs were satisfied with the “MOVEdiabetes” study. Additionally, the majority of the participants perceived the programme as appropriate within primary diabetes care in Oman. The fact that this intervention was delivered in a primary care setting may have enhanced intervention implementation and acceptance as this setting has been reported as being effective for PA promotion [16–19]. Primary health care is considered as one of seven best investments by the Global Advocacy for PA [20, 21]. It is therefore reassuring for interested researchers to upscale the current study or develop similar PA interventions within diabetes clinical settings.

Opportunities to ask questions and feedback were well received by both the participants and POs. In fact, the information received was perceived as ‘more than necessary’ or ‘far too much’ by more than half of the participants. A future assessment may be needed to explore which aspects of the project require more information.

The communications in the “MOVEdiabetes” study were accessible and flexible throughout the study period. Participants had options for interactive communications with their peers and/or POs through WhatsApp or face to face contacts in the health centres within the scheduled visits to diabetes clinics. This may have initiated a positive social atmosphere for PA support [22]. This feature may have contributed to their willingness to recommend the project to others and to their subjective perceptions of improvements in their PA behaviour. Findings from the literature confirmed the positive effects of psycho-social influences namely self-efficacy and social support on levels of PA [22–27]. However, future studies may consider exploring robust ways for effective and sustainable communications including providing information and feedback in promoting PA in diabetes care.

The intervention components used in the “MOVEdiabetes” study were a practical translation of the recommendations from the formative work carried out to inform the PA intervention design [10, 13]. This study demonstrated that the “MOVEdiabetes” intervention components (face to face personalised PA consultations, pedometer and WhatsApp use) within routine diabetes primary care were satisfactory, appropriate and acceptable by the majority of the participants and POs. However, some participants perceived the longevity, defined as the period of time within which the device (pedometers) was operating well, as ‘very poor’ or ‘poor’ (device stopped working/recording the steps taken/day). This may indicate the need to consider devices with better quality and longer longevity especially with the current available pedometers in the market. Most importantly, participants need to understand the limitations of pedometers including risk of falling and damage (For example pedometer use in swimming).

It is evident that the WhatsApp, PA consultations and pedometer use was highly rated by participants. The POs on the other hand, gave more positive ratings for delivering the consultations, pedometers and then WhatsApp use. POs may value clinical based settings for consultations as a normal part of their daily work and may not have time to engage in additional (outside the clinic) communications [4, 28, 29]. This challenge was possibly diluted by the fact that the project was managed by a team of four members in each of the health centres who took turns to give feedback to participants. On the other hand, the participant/patients may have considered the WhatsApp communications as an additional flexible tool to discuss their health condition with their health care providers. This may have facilitated the establishment of a better patient-provider relationship reflected in the high participants’ satisfaction on the opportunities to ask questions and getting answers/feedback reported earlier. The positive effects of using the WhatsApp phone application in promoting PA has been reported in few studies [30]. However, more information is required on the long-term use of phone and text applications on promoting healthy behaviours.

Two research perspectives were identified as challenging by the participants and POs. Firstly, the multiple questionnaires (GPAQ, self-efficacy, social support, general well-being and exit questionnaires) used in this study were viewed as too long and time consuming. However, it is important to note that these were used for research purposes and may not be used within the common routine diabetes clinics. Future simpler versions of those questionnaires may be warranted for service evaluation purposes if these were to be integrated within the routine diabetes primary care. Secondly, delivery of the PA intervention was linked to pre-scheduled visits to diabetes clinics. Due to the dynamic and busy nature of the diabetes primary clinics as reported by the POs, future interventions may test the effectiveness of “stand alone” PA clinics that patients could be referred to vs the integrative “MOVEdiabetes” approach [31]. However, the fact that most participants found coming to the clinic for visits easy, may be attributed to the integrative approach adapted in the current study.

Similar to many studies in nearby countries, [32–34] hot weather was cited as a barrier by responders from the “MOVEdiabetes” study indicating the importance of discussing options for indoor PA and/or weather friendly timings for PA. However, addressing extreme weather conditions in promoting PA is under reported [35, 36].

Finally, participants highlighted the desire for advice on diet as an adjunct to PA and also for similar PA promotion projects to be available for all (the general population). These recommendations are of direct relevance to the National Health Policy Priorities in Oman [37]. To promote the health awareness of the community and establish a culture of healthy lifestyles" [37, 38].

With respect to the POs, qualitative data indicates a desire for more training on PA behaviour change techniques and measurement tools [4]. This may be essential for the continuation of the capacity building activities in PA across health care professions.

Challenges to delivering the intervention by the POs were similar to those reported in the literature e.g. the physical and logistical constraints within the structure of primary health care (e.g. small rooms and lack of space) [3]. Future extension of this project could explore the optimal approaches to re-structure and organise the routine diabetes clinics to make them friendly to PA promotion for both patients and health care providers.

### Study limitations

The interviewer led approach may have discouraged the participant from giving negative comments and more work may be needed to explore views and perceptions using anonymous approaches. Also, bias cannot be excluded as answers to the open ended questions were limited to those who responded and it is possible that non-responders may have not been interested or entirely satisfied.

Moreover, the current exit surveys looked at perceptions from the intervention group only; future studies may consider exploring views from the comparison groups too. This would confirm if the perceptions on the study programme were actually different between the study groups e.g. perceptions of changed behaviours.

## Conclusions

The “MOVEdiabetes” study was perceived as satisfactory, appropriate and suitable. Overall, the suggested alterations to the PA intervention (inclusion of advice on diet, PA trainings, shorter PA evaluative tools, integration of PA in the current HIS, and links to community resources) are hoped to lead to a sustainable PA service within the current primary health care setting that could be made available for the general population.

## Supplementary information

**Additional file 1:** “MOVEdiabetes” End of Study Questionnaire - Participant

**Additional file 2:** “MOVEdiabetes” End of Study Questionnaire - Project officer

**Additional file 3:** Quotations from the participant (open questions exit survey)

**Additional file 4:** Quotes from the POs (open questions exit survey)

## Data Availability

Data generated from this study is not available for public use. However it is available from the corresponding author on reasonable request and approvals from Oman Ministry of Health.

## Abbreviations

PA: Physical activity
T2D: Type 2 diabetes
IGP: Intervention Group Participants
POs: Project Officers
IG: Intervention group
CG: Comparison group
GPAQ: Global physical activity questionnaire
(Scot-PASQ): The Scottish Physical Activity Screening Questionnaire
