# Supplementary material for: Acceptability of the “MOVEdiabetes” physical activity intervention in diabetes primary care settings in Oman: findings from participants and practitioners
Source: BMC Public Health. 2020 Jun 8;20:887. doi: 10.1186/s12889-020-09029-1 (PMC7281938; doi:10.1186/s12889-020-09029-1)
Supplement: Supplementary file 1 — Additional file 1: “MOVEdiabetes” End of Study Questionnaire - Participant [file 12889_2020_9029_MOESM1_ESM.pdf]

"MOVEdiabetes"
End of Study Questionnaire
Participant

Thank you for participating in the "MOVEdiabetes" study over the past 12 months. Please take a few minutes to complete this evaluation questionnaire **by placing a tick in the relevant box and answering questions as required** –your replies may help us improve physical activity services in routine diabetes care

Please note:

- Your answers are being assessed independently.
- Your answers will be kept anonymous and confidential.

1. Overall, how satisfied were you with the "MOVEdiabetes" project?				
Very dissatisfied	Quite dissatisfied	Neither satisfied nor dissatisfied	Quite satisfied	Very satisfied

2. Do you feel you received enough information about the project at the start?				
Far too little	Not enough information	Sufficient information	More information than was necessary	Far too much information

3. Which aspects of the project do you wish you'd had more information on?

4. Did you have enough opportunity to ask questions during the project?				
Not at all	Rarely	Every once in a while	Sometimes	Very often

5. Were your questions answered to your satisfaction?				
Not at all	Rarely	Every once in a while	Sometimes	Yes, completely

6. Which of the following most closely describes the number of face-to-face consultations you received?				
No visits	1 visits	2 visits	3 visit	More than 3

7. How likely are you to recommend “MOVEdiabetes” to other people?				
Very unlikely	Quite unlikely	Neither likely nor unlikely	Quite likely	Very likely

8. How did you find coming up to the health centre for your appointments?				
Very difficult	Quite difficult	Neither easy nor difficult	Quite easy	Very easy

9. Having taken part, do you think this program is appropriate in diabetes care?				
No, not at all appropriate	Quite inappropriate	Neither appropriate or inappropriate	Quite appropriate	Very appropriate

10. was your physical activity behaviour change acceptable?				
Not at all acceptable	Not very acceptable	Neither acceptable or unacceptable	Quite acceptable	Very acceptable To a

11. What were the challenges of taking part in this project?

12. What were the barriers to increasing your physical activity behaviour?

13. Please rate the consultations you received					
	Very poor	poor	acceptable	good	Very good
Content					
Relevance					
Duration per consultation					
Frequency					

14. Please rate using pedometers					
	Very poor	Poor	Neither poor nor good	Fairly good	Very good
Length of device use					
Importance to diabetes management					
Wearing it (put it on and off)					
Usefulness					

15. Please rate the WhatsApp communication you received					
	Very poor	Poor	Neither poor nor good	Fairly good	Very good
Content					
Relevance					
Time required					

Frequency of messages					
Supportiveness					

16. Gender	
Male	Female

17. Please feel free to make any other general comments in the space below:

Thank you for your participation in the “MOVEdiabetes” study and for completing this survey.
