# Supplementary material for: Acceptability of the “MOVEdiabetes” physical activity intervention in diabetes primary care settings in Oman: findings from participants and practitioners
Source: BMC Public Health. 2020 Jun 8;20:887. doi: 10.1186/s12889-020-09029-1 (PMC7281938; doi:10.1186/s12889-020-09029-1)
Supplement: Supplementary file 2 — Additional file 2: “MOVEdiabetes” End of Study Questionnaire - Project officer [file 12889_2020_9029_MOESM2_ESM.pdf]

"MOVEdiabetes"
End of Study Questionnaire
Project officer

Thank you for participating in the "MOVEdiabetes" study over the past 12 months. Please take a few minutes to complete this evaluation questionnaire **by placing a tick in the relevant box and answering questions as required** –your replies may help us improve physical activity services in routine diabetes care

Please note:

- Your answers are being assessed independently.
- Your answers will be kept anonymous and confidential.

1. Overall, how satisfied were you with the "MOVEdiabetes" project?				
Very dissatisfied	Quite dissatisfied	Neither satisfied nor dissatisfied	Quite satisfied	Very satisfied

2. Do you feel you received enough training about the project at the start?				
Far too little	Not enough information	Sufficient information	More information than was necessary	Far too much information

3. Which aspects of the project do you wish you'd had more information on?

4. Did you have enough opportunity to ask questions during the project?				
Not at all	Rarely	Every once in a while	Sometimes	Very often

5. Were your questions answered to your satisfaction?				
Not at all	Rarely	Every once in a while	Sometimes	Yes, completely

6. Which of the following most closely describes the number of face-to-face consultations you conducted?				
No visits	1 visits	2 visits	3 visit	More than 3

7. Having taken part, do you think this programme is appropriate in diabetes care?				
No, not at all appropriate	Quite inappropriate	Not sure	Quite appropriate	Very appropriate

8. What were the challenges of taking part in this project?

9. Please rate the consultations you conducted					
	Very poor	Poor	Acceptable	Good	Very good
Content					
Relevance					
frequency					

10. Please rate the use of pedometers as physical activity self-monitoring tool					
	Very poor	Poor	Acceptable	Fairly good	Very good
Usefulness					
Relevance to diabetes management					

11. Please rate the WhatsApp communication you were involved in					
	Very poor	Poor	Acceptable	Fairly good	Very good
Content					
Relevance					
Time required					
Frequency of messages					

12. How suitable is this project to the current diabetes primary care?					
	Not at all suitable	Not very suitable	Not sure	Quite suitable	Very suitable
Consultations					
Pedometers					
Personal PA diaries					
WhatsApp					

13. Gender	
Male	Female

14. Are you (1-dietician, 2- doctor, 3- nurse, 4-health educator, 5-other (specify)

15. Please feel free to make any other general comments in the space below:

Thank you for your participation in the “MOVEdiabetes” study and for completing this survey.
