# Supplementary material for: Acceptability of the “MOVEdiabetes” physical activity intervention in diabetes primary care settings in Oman: findings from participants and practitioners
Source: BMC Public Health. 2020 Jun 8;20:887. doi: 10.1186/s12889-020-09029-1 (PMC7281938; doi:10.1186/s12889-020-09029-1)
Supplement: Supplementary file 3 — Additional file 3: Quotations from the participant (open questions exit survey) [file 12889_2020_9029_MOESM3_ESM.docx]

Quotations from the participant (open questions exit survey):

| Themes | Responses (quotes) | Participants’ health centre | Number of participants (%) |
| --- | --- | --- | --- |
| Q1: Aspects of the project where more information was needed | | | |
| No response |  |  | 47 (56.1) |
| Types of exercises in diabetes | What type of exercise is suitable for patients with diabetes?  Can patients with diabetes do aerobic exercises?  What about resistance exercises in diabetes management?  Is walking sufficient to control diabetes?  What exercises we can do?  What is the best exercise for patients with diabetes?  Is running good for patients with diabetes?  How can I exercise if my sugar is high?  Is walking an enough exercise?  What about playing football, is it enough one a week?  What can I do to increase my physical activity?  What about swimming?  I like to go to the gym, but not sure of the physical activity machines to use?  Can I walk on the beach?  Is playing basketball a good exercise?  What exercise is suitable at workplace?  How to exercise in hot weather?  For busy house wife like me, what can I do to increase my physical activity? | HC1  HC3  HC2  HC2  HC4  HC1  HC1  HC1  HC4  HC3  HC1  HC1  HC3  HC3  HC1  HC3  HC2  HC1 | 18 (22) |
| Use of accelerometers | Can we attach the accelerometers on the arm?  What is the purpose of the accelerometers?  I don’t like the skin irritation from accelerometers, can I remove it?  Can we use then while swimming?  Does it carry any side effects?  Why put the accelerometers on upper thigh?  Why it is used in this study?  Why some patients are wearing them and some are not?  Can we keep them by ourselves?  Can we travel with these on?  Can we pray with these on? | HC3  HC1  HC1  HC1  HC4  HC3  HC1  HC1  HC3  HC2  HC2 | 11 (13.4) |
| PA options in presence of comorbidities | What can I do if I have joint pain?  What is the best physical activity in case of high blood pressure?  I have glaucoma, can I exercise?  How can I exercise with back pain?  I have muscular pain when I perform any physical activity, what can I do?  What is PA recommendations for patients with diabetes on insulin? | HC2  HC3  HC2  HC1  HC3  HC1 | 6 (8.5) |
| Q2: Challenges of taking part in this project | | | |
| No response |  |  | 61 (74.4) |
| Nature of PA measurment tools | The questionnaires are too long and time consuming  The device attached on my thigh is not comfortable  I get confused sometimes if you give me too much instructions  Accelerometers are not comfortable  Difficult to respond to the long questionnaires  Long and difficult questionnaires  I don’t understand all the questions in the questionnaires  I cannot wear accelerometer during sleeping time  Questions on PA are difficult  Too much instructions  Difficult to follow all PA guidelines  Too many papers in the questionnaire  Consultations are long due to exhausting questionnaires | HC4  HC1  HC3  HC3  HC2  HC1  HC2  HC1  HC4  HC2  HC1  HC2  HC1 | 13 (15.9) |
| Time limitation | I don’t have time to attend the PA consultations  Time is not enough for my diabetes care and PA advice  I am short of time  Time is short  Time is not enough  Difficult to add PA in diabetes care, too busy and no time  All staff are busy. They have no time for PA  Diabetes clinics are too busy | HC2 | 8 (9.8) |
| Q3: Barriers to increasing physical activity behaviour other | | | |
| No response |  |  | 46(56.1) |
| Weather | It is too hot outside, I can’t walk  What can we do in summer times  Oman is very hot in the summer  I can’t walk if it’s too hot  What can I do if the weather is too hot  Sometimes I try to walk outside, but I can’t due to extreme heat  I don’t like walking in the summer  The weather is not friendly  It is inconvenient to do PA in hot weather  The weather is not suitable  We need to think of alternative in door physical activities if its hot outside  If its hot outside, it’s better to avoid walking especially when comorbidities exist  It’s hot throughout the year  There is no way to avoid walking in the summer but one should take care  How can I play football if the weather is bad  What can I do if it’s hot outside? | HC4  HC3  HC1  HC4  HC3  HC4  HC1  HC1  HC1  HC4  HC4  HC4  HC3  HC2  HC2  HC4 | 16(19.5) |
| Lack of time | I have no time for physical activity  Time is not enough  I work for a long time and become tired at the end of the day  We are too busy  I can’t make time for physical activity  I am busy with kids all the time  I work two shifts most days of the week  I am too tired to do any physical activity due to lack of time  I have many work responsibilities that prevents me from performing PA  Time is not enough especially in weekends  I have to make time for physical activity  Time is short  Time management is difficult for physical activity  I am busy with kids at home I have no time for physical activity  Most of the times I am busy with work, family and friends | HC1  HC4  HC3  HC3  HC2  HC1  HC1  HC1  HC4  HC1  HC1  HC3  HC4  HC4  HC2 | 15(18.3) |
| Pain | I can’t exercise due to pain  I have pain in my back  My feet heart when I exercise  I have knee pain  When I walk I get muscular pain. | HC2  HC2  HC3  HC1  HC4 | 5(6.1) |
| Q4: General comments | | | |
| No response |  |  | 62(75.6) |
| Diet advice | I suggest to add diet advice  What about diet in diabetes care?  What can I eat when I exercise?  Can we have more advice on diet?  Who can give us more diet advice?  I like to perform PA but not sure of what to eat?  How can I lose weight from PA and diet?  I feel so tired to do any PA if I don’t eat a proper meal. What can I do?  Is it OK to exercise and cut down sugars?  I don’t understand what to eat if I exercise  It is important to know the proper diet  Why not including similar intervention to promote healthy diet  I want to know more about the proper food in diabetes  How can PA work without proper diet advice?  I suggest to include diabetes diet in future studies | HC2  HC1  HC3  HC4  HC3  HC1  HC2  HC2  HC3  HC1  HC3  HC2  HC2  HC1  HC4 | 15(18.3) |
| Project sustainability | Keep the project don’t stop  I want to keep pedometers  Can we continue the WhatsApp communications | HC3  HC4  HC1 | 3 (3.7) |
| Similar project for children and all | Develop similar projects for children  Develop similar projects for the public | HC3  HC1 | 2(2.4) |
