# Supplementary material for: Acceptability of the “MOVEdiabetes” physical activity intervention in diabetes primary care settings in Oman: findings from participants and practitioners
Source: BMC Public Health. 2020 Jun 8;20:887. doi: 10.1186/s12889-020-09029-1 (PMC7281938; doi:10.1186/s12889-020-09029-1)
Supplement: Supplementary file 4 — Additional file 4: Quotes from the POs (open questions exit survey) [file 12889_2020_9029_MOESM4_ESM.docx]

Quotes from the POs (open questions exit survey):

| Themes | Responses (quotes) | POs number | Numbers (%) |
| --- | --- | --- | --- |
| Q1: Topic which require more information | | | |
| No response |  |  | 4 (25) |
| physical activity Behaviour change techniques | We need more physical activity training especially on the behaviour change techniques  I think the behaviour change techniques are difficult to understand, we need more training  It’s difficult to translate the behaviour change techniques from English to Arabic. A training manual may be needed  What are the behaviour change techniques and how to apply them in promoting physical activity in diabetes care?  Where can we get more training on applying the physical activity behaviour change techniques in diabetes care?  Can we implement behaviour change techniques in all lifestyle initiatives?  Where can we get an extensive training on behaviour change techniques?  I suggest to have master trainers in physical activity behaviour change techniques | PO1  PO4  PO1  PO8  PO5  PO12  PO9  PO2 | 8 (50) |
| physical activity measurement tools | Can we attach replace accelerometers with other devices?  What is the purpose of the questionnaires?  How can we shorten the physical activity questionnaire?  Can we use a simpler physical activity questionnaires? |  | 4 (25) |
| Q2: Challenges to delivering the intervention | | | |
| Physical challenges | No dedicated room/space  Busy clinics  No physical activity facilities  Lack of staff  Staff turnover  Consultation rooms are small  The setting in the clinics is not welcoming as there are too many furniture  Diabetes clinic require more supportive physical activity tools  There is no proper waiting areas for patients and their families to wait | PO3  PO2  PO16  PO3  PO6  PO7  PO1  PO2  PO6 | 9 (56.2) |
| Logistical challenges | Consultations are too long sometimes  Long questionnaires  Managing appointments was difficult  Handling accelerometers was difficult  Participant don’t attend on time  It is sometimes difficult to demonstrate effective physical activity without appropriate tools  Adherence to appointments | PO12  PO16  PO5  PO10  PO3  PO10  PO8 | 7 (43.8) |
| Q3: General comments | | | |
| No response |  |  | 2(12.5) |
| Sustaining the intervention | WhatsApp communications may be useful for future physical activity interventions  Include physical activity in the Health information system  Can we keep the pedometers?  This project can be a good start for integrating physical activity in primary health care  Can we continue the physical activity consultations  We need to train everyone in the health centre  All staff working in the health centres may benefit from training on PA consultations  Keep the same staff don’t transfer or mobilize  Implement this project in all health centres  This project needs to be monitored and evaluated similar to all other primary care public health programmes  A specific budget needs to be allocated to maintain physical activity services in diabetes care  Involve all stakeholders | PO8  PO3  PO4  PO1  PO7  PO1  PO10  PO16  PO12  PO7  PO3  PO11 | 12(75) |
| physical activity facilities | We need information on the available physical activity facilities in the nearby community  Where can we refer the patient to for physical activity? | PO5  PO6 | 2(12.5) |
